# Association of daytime napping frequency and schizophrenia: a bidirectional two-sample Mendelian randomization study

**DOI:** 10.1186/s12888-022-04431-y

**Published:** 2022-12-13

**Authors:** Jun Ma, Chen Jin, Yan Yang, Haoqi Li, Yi Wang

**Affiliations:** 1 Department of Epidemiology and Biostatistics, School of Public Health and Management, Wenzhou Medical University, Wenzhou, China; 2 Institute of Aging, Key Laboratory of Alzheimer’s Disease of Zhejiang Province, Wenzhou Medical University, Wenzhou Medical University, Wenzhou, Zhejiang China

**Keywords:** Mendelian randomization, Daytime napping, Schizophrenia, Single nucleotide polymorphism, Genome-wide association studies

## Abstract

**Background:**

The bidirectional causal association between daytime napping frequency and schizophrenia is unclear.

**Methods:**

A bidirectional two-sample Mendelian randomization (MR) analysis was conducted with summary statistics of top genetic variants associated with daytime napping frequency and schizophrenia from genome-wide association studies (GWAS). The single nucleotide polymorphisms (SNPs) data of daytime napping frequency GWAS came from the UK Biobank (*n* = 452,633) and 23andMe study cohort (*n* = 541,333), while the schizophrenia GWAS came from the Psychiatric Genomics Consortium (PGC, 36,989 cases and 113,075 controls). The inverse variance weighted (IVW) analysis was the primary method, with the weighted median, MR-Robust Adjusted Profile Score (RAPS), Radial MR and MR-Pleiotropy Residual Sum Outlier (PRESSO) as sensitivity analysis.

**Results:**

The MR analysis showed a bidirectional causal relationship between more frequent daytime napping and the occurrence of schizophrenia, with the odds ratio (OR) for one-unit increase in napping category (never, sometimes, usually) on schizophrenia was 3.38 (95% confidence interval [CI]: 2.02–5.65, *P* = 3.58 × 10^–6^), and the beta for the occurrence of schizophrenia on daytime napping frequency was 0.0112 (95%CI: 0.0060–0.0163, *P* = 2.04 × 10^–5^). The sensitivity analysis obtained the same conclusions.

**Conclusion:**

Our findings support the bidirectional causal association between more daytime napping frequency and schizophrenia, implying that daytime napping frequency is a potential intervention for the progression and treatment of schizophrenia.

**Supplementary Information:**

The online version contains supplementary material available at 10.1186/s12888-022-04431-y.

## Background

Schizophrenia is a complex and debilitating psychiatric disorder [1] that has been regarded as one of the world's most severe and disabling illnesses [2], with symptoms typically appearing in late adolescence or early adulthood [3]. Approximately 21 million people worldwide suffer from schizophrenia [3]. The lifetime prevalence of schizophrenia is roughly 1% [4], with the most detailed prevalence study conducted in Finland yielding a result of 0.87%, but the variation is up to five times higher around the world [5]. Schizophrenia is characterized by "positive symptoms" of hallucinations, delusions, and verbal confusion, "negative symptoms" of decreased motivation and expressivity, and cognitive deficits involving impaired executive functioning, memory, and mental processing speed [6].

Daytime napping is a cross-cultural phenomenon that occurs throughout human life [7], particularly common in countries with Mediterranean cultures and some non-Mediterranean countries, such as the USA [8]. Daytime napping can reduce fatigue [9], enhance emotional processing[9], facilitate the formation of long-term memory [10], and improve subjective and behavioral measures, as well as mood and subjective sleepiness levels [7], which appears to be a beneficial intervention to facilitate the recovery and mitigate the negative physical and cognitive effects of sleep deprivation [11]. Daytime napping of about 40 min can improve declarative memory performance in patients with schizophrenia [12].

However, frequent napping was associated with a range of adverse outcomes, including hypertension [13], vascular disease [14], depression disorder [15], and diabetes [16], especially cognitive decline in psychiatric disorders[9]. A longer daytime nap was typically associated with more significant cognitive decline and a higher risk of cognitive impairment in older men [17]. Sleep has been identified as a process of re-integrating information in the brain, and cognitive dysfunction is a common feature in patients with schizophrenia [12]. The study by Michael Wainberg et al., showed a significant increase in daytime napping frequency among schizophrenia patients [18], but the bidirectional causality between the more daytime napping frequency and schizophrenia is unclear. The bidirectional causality between more frequent daytime napping and schizophrenia could be biased due to possible confounding factors, which are difficult to confirm by observational epidemiological methods, such as sleep duration, insomnia, daytime sleepiness and wake-up time.

Mendelian randomization (MR) is a statistical approach that uses genetic variation, i.e., single nucleotide polymorphisms (SNPs), as instrumental variables (IVs) to control confounding factors [19]. MR relies on the random assortment of genetic variation during meiosis, leading to a random distribution of genetic variation in the population [20]. MR can overcome the influence of confounding factors and has a superior advantage in the face of causal inference of exposure-outcome due to the genotype being determined before birth [21]. MR uses summary statistics from genome-wide association studies (GWAS) of large amounts of genetic variation to extract SNPs associated with exposure and outcome variables, which can further reveal clear causal relationships between the exposure and outcome [22]. Therefore, this study aims to infer the bidirectional causality between more frequent daytime napping and schizophrenia by using two-sample MR to provide more reliable evidence.

## Methods

### Study design

**Fig. 1:**
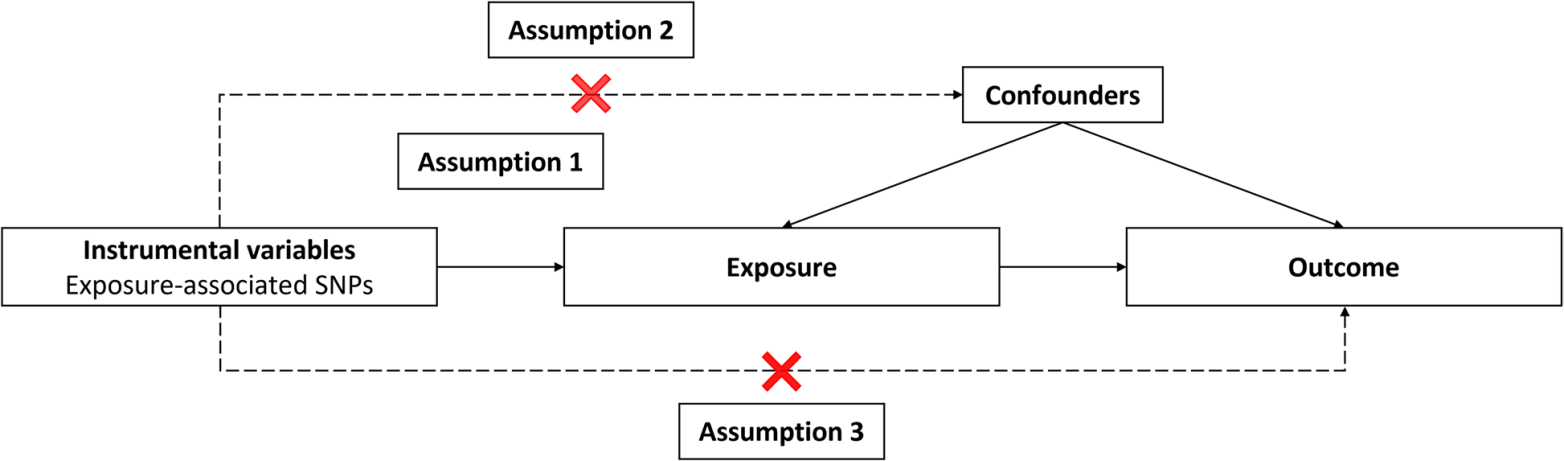
Mendelian randomization instrumental variables assumptions

The MR approach is based on three main assumptions (Fig. 1). Firstly, genetic variants used as IVs are strongly associated with exposure (Assumption 1). Secondly, genetic variants are independent of confounding factors (Assumption 2). Thirdly, horizontal pleiotropy should be avoided (Assumption 3). Horizontal pleiotropy means that genetic variation affects multiple traits through independent pathways, it occurs when the genetic variant influences the outcome outside of its effect on the exposure in Mendelian randomization [22].

### Genetic associations with daytime napping frequency

The daytime napping frequency summary statistics came from the GWAS study of the UK Biobank (*n* = 452, 633) and was replicated and validated in the 23andMe cohort (*n* = 541,333) [23]. The UK Biobank is a population-based cohort that recruited 500,000 40–69 years old participants during 2006–2010 and obtained a wide range of phenotypes and health information, such as biometric measurements, biomarkers in blood and urine, and lifestyle indicators [24]. In this study, a total of 123 SNPs reached genome-wide significance (*P* < 5 × 10^–8^) and explained 1.1% of the variance in daytime napping frequency. 23andMe is a direct-to-consumer genetic testing company. These 123 SNPs were replicated and validated in the 23andMe study cohort (*n* = 541,333), of which 94 SNPs were still present in the 23andMe cohort. For the genetic variant replication of daytime napping frequency in the 23andMe cohort, participants were asked the question "How many days per week do you take a daytime nap? (15 min or more)” and chose one from the following four options: "never/rarely (0 or 1)", "sometimes (2 to 5)", "usually (6 or 7)", "Tend not to answer". Since the population of the subsequent GWAS study on schizophrenia may partially overlap with that of the UK Biobank study, the 94 SNPs that were replicated and validated in the 23andMe study cohort were used as IVs in this study for subsequent analyses to avoid the bias of winner's curse caused by the overlapping samples [25].

### Genetic associations with schizophrenia

The schizophrenia summary statistics came from a GWAS study by the Psychiatric Genomics Consortium (PGC) that constructed the genome-wide genotype data of 49 ancestry-matched, non-overlapping case–control samples (34,241 cases and 45,604 controls) and three family-based European ancestry samples (1,235 parent-affected-offspring trios) [26]. The GWAS study identified 128 independent SNPs associated with schizophrenia, of which 108 met genome-wide significance(*P* < 5 × 10^–8^). The summary statistics is available in the IEU OpenGWAS project database (https://gwas.mrcieu.ac.uk/) with the GWAS ID "ieu-b-42".

### Instrumental variables selection

We extracted the SNPs that met genome-wide significance from the GWAS summary statistics of daytime napping frequency and schizophrenia, respectively. To ensure the reliability of the findings, we performed the PLINK clumping method with a stringent clumping threshold (r^2^ < 0.001, kb = 10,000) to ensure that SNPs in residual linkage-disequilibrium (LD) within a particular window were pruned to assess the bias caused by residual LD of genetic variants. We calculated the *F*-statistic for IVs, and an *F* > 10 means sufficient for MR analysis in general [22]. In addition, we selected appropriate proxy SNPs (r^2^ > 0.8) when exposure-associated SNPs were not present in the outcome summary statistics and removed the SNPs with palindromic structure during the analysis.

### MR analyses

WE applied the inverse-variance weighted (IVW) method as the primary MR analysis to estimate the bidirectional causal relationship between daytime napping frequency and schizophrenia. To assess the robustness of the IVW analysis results, we performed additional tests for horizontal pleiotropy to detect heterogeneous outcomes, including the Cochran’s *Q* statistic test and the MR Egger intercept test. We also performed (i) the weighted median method, which allows SNPs with the more significant beta to contribute more to the estimate and can be derived by weighting the contribution of each SNP by the inverse variance [27]; (ii) MR robust adjusted profile score (MR-RAPS), which can give a robust inference for MR analysis with weak instrumental variables, especially when both exposure and outcome are complex traits [28]; (iii) MR Pleiotropy Residual Sum and Outlier (MR-PRESSO) test, which detects and corrects for horizontal pleiotropic outliers [29] and (iv) Radial MR, which improves detection of outliers in IVW and MR-Egger analysis by radial plots [30] as sensitivity analyses afterward.

The bidirectional two-sample MR analysis, horizontal pleiotropy tests, and sensitivity analyses were performed with the *TwoSampleMR* package (version 0.5.6), *mr. raps* (version 0.2), *Radial MR* (version 1.0), and *MR-PRESSO* (version 1.0) in R program (R Foundation for Statistical Computing, version 4.1.2).

## Results

### Instrumental variables selection and mendelian randomization analysis

**Fig. 2:**
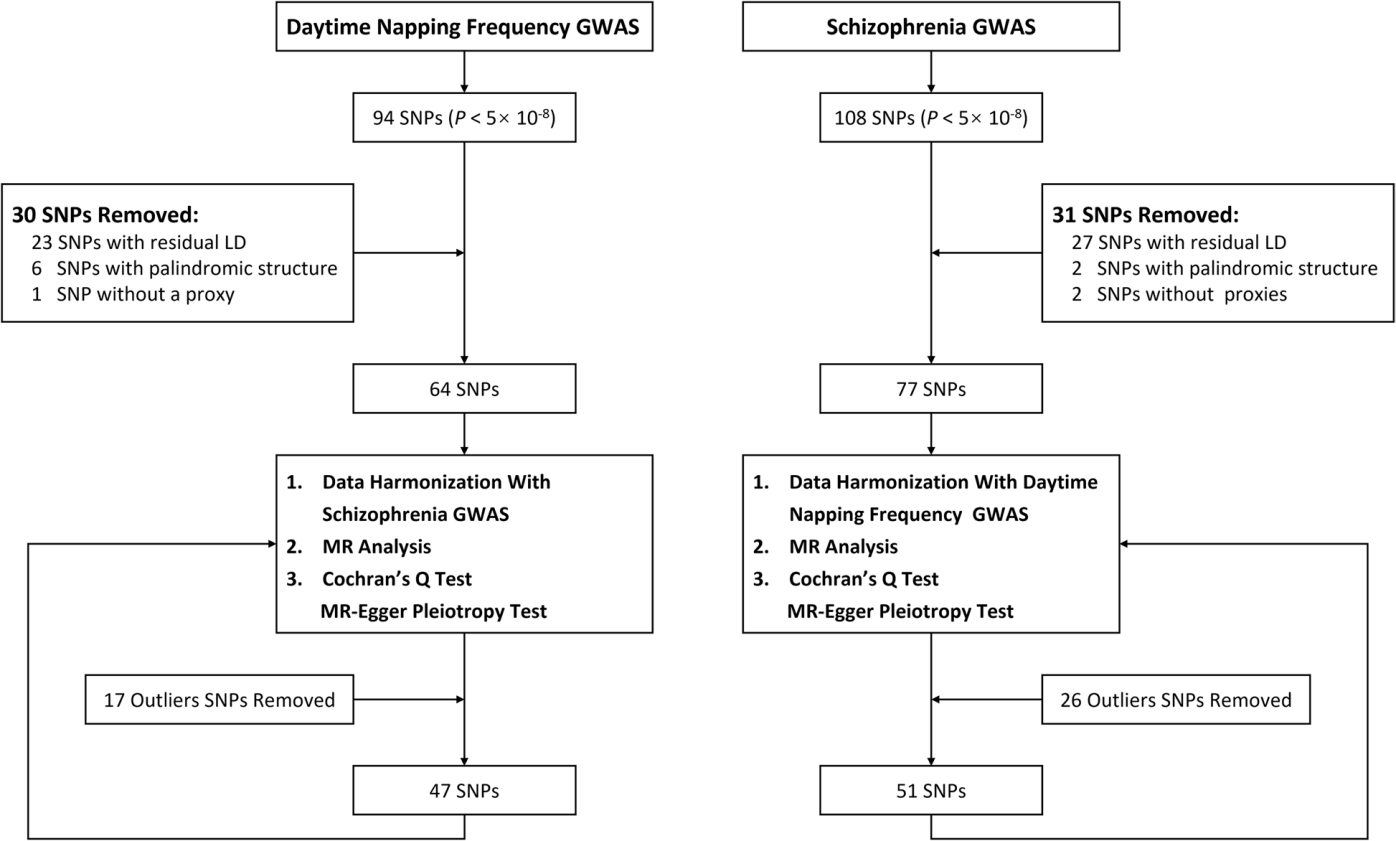
The flow chart of Mendelian randomization. Abbreviations: GWAS, genome-wide association studies; SNP, single nucleotide polymorphism; LD, linkage-disequilibrium; MR, Mendelian randomization

The flow chart for the selection of IVs and MR analysis is shown in Fig. 2. We extracted 94 and 108 SNPs as the initial IVs from the GWAS summary statistics of daytime napping frequency and schizophrenia, respectively. A total of 64 and 77 SNPs associated with daytime napping frequency and schizophrenia were included in the follow-up MR analysis after PLINK clumping progress, selection of appropriate proxy SNPs and exclusion of SNPs with palindromic structure, respectively. The Cochran’s *Q* statistic test suggested significant heterogeneity, and MR Egger intercept test suggested no significant horizontal pleiotropy (Table 1 and Table 2). After the detection of outliers by Radial MR, we performed MR analysis again with 47 and 51 SNPs (Supplementary Table S1 and Table S2) associated with daytime napping frequency and schizophrenia as the final IVs, respectively.

**Table 1:** Causal estimates for daytime napping frequency on schizophrenia by Mendelian randomization. Abbreviations: SNP, single nucleotide polymorphism; IVW, inverse variance weighted; MR-RAPS, Mendelian randomization robust adjusted profile score; MR-PRESSO, Mendelian randomization pleiotropy residual sum and outlier

Exposure	Outcome	SNPs (n)	Cochran’s *Q P*-value	MR-Egger pleiotropy test *P*-value	Methods	OR (95% CI)	*P*-value
Daytime Napping Frequency	Schizophrenia	64	1.04E-19	0.27	IVW	3.48(1.54,7.85)	2.65E-03
					Weighted median	3.30(1.52,7.15)	2.56E-03
					MR-RAPS	4.50(2.92,6.93)	8.50E-12
					MR-PRESSO	3.68(1.65,8.20)	2.19E-03
					Radial MR	3.67(1.65,8.18)	1.48E-03
Daytime Napping Frequency (Without outliers)	Schizophrenia	47	0.154	0.68	IVW	3.38(2.02,5.65)	3.58E-06
					Weighted median	3.49(1.67,7.27)	8.57E-04
					MR-RAPS	3.55(2.18,5.78)	3.67E-07
					MR-PRESSO	3.38(2.02,5.65)	2.97E-05
					Radial MR	3.37(2.01,5.63)	3.81E-06

**Table 2:** Causal estimates for schizophrenia on daytime napping frequency by Mendelian randomization. Abbreviations: SNP, single nucleotide polymorphism; IVW, inverse variance weighted; MR-RAPS, Mendelian randomization robust adjusted profile score; MR-PRESSO, Mendelian randomization pleiotropy residual sum and outlier

Exposure	Outcome	SNPs (n)	Cochran’s *Q P*-value	MR-Egger pleiotropy test *P*-value	Methods	beta (95% CI)	*P*-value
Schizophrenia	Daytime Napping Frequency	77	4.05E-40	0.21	IVW	0.0095(0.0012,0.0178)	2.50E-02
					Weighted median	0.0106(0.0035,0.0176)	3.37E-03
					MR-RAPS	0.0102(0.0065,0.0138)	5.88E-08
					MR-PRESSO	0.0090(0.0008,0.0172)	3.18E-02
					Radial MR	0.0090(0.0008,0.0172)	3.18E-02
Schizophrenia (Without outliers)	Daytime Napping Frequency	51	0.173	0.67	IVW	0.0112(0.0060,0.0163)	2.04E-05
					Weighted median	0.0114(0.0043,0.0185)	1.67E-03
					MR-RAPS	0.0115(0.0066,0.0164)	3.51E-06
					MR-PRESSO	0.0112(0.0060,0.0163)	9.00E-05
					Radial MR	0.0112(0.0060,0.0163)	2.06E-05

### Causality of daytime napping frequency and schizophrenia

The *F*-statistic for all IVs associated with daytime napping frequency and schizophrenia were > 10, which effectively avoided weak IVs bias. The IVW method showed a bidirectional causal relationship between daytime napping frequency and the occurrence of schizophrenia (Table 1 and Table2), with the odds ratio (OR) for one-unit increase in napping category (never, sometimes, usually) on schizophrenia was 3.48 (95% confidence interval [CI]: 1.54–7.85, *P* = 2.65 × 10^–3^; without outliers: OR = 3.38, 95% CI: 2.02–5.65, *P* = 3.58 × 10^–6^) and the beta for the occurrence of schizophrenia on daytime napping frequency was 0.0095 (95%CI: 0.0012–0.0178, *P* = 2.50 × 10^–2^; without outliers: beta = 0.0112, 95%CI: 0.0060–0.0163, *P* = 2.04 × 10^–5^). The weighted median, MR-RAPS, Radial MR, MR-PRESSO methods obtained consistent conclusions (Tables 1 and 2).

## Discussion

Schizophrenia is frequently regarded as one of the most serious mental illnesses[2], with patients having a life expectancy approximately 15 years shorter than the general population and a 5% to 10% lifetime risk of suicide[4]. Schizophrenia is one of the most disabling of all disorders in both developing and developed countries and is associated with reduced social connectedness, lower employment rates, and impaired independent living[1]. Therefore, the development of effective strategies to prevent schizophrenia and reduce or avoid risk factors for schizophrenia is important to improve population health. We validated IVs from the GWAS study and performed the MR method to strengthen the bidirectional causal inference between the daytime napping frequency and schizophrenia. Results suggested a potential bidirectional causal association between more frequent daytime napping and the occurrence of schizophrenia.

Schizophrenia is accompanied by sleep abnormalities and is associated with more serious psychotic symptoms and worse clinical outcomes [31]. Sleep abnormalities in patients with schizophrenia include insomnia, nightmares, and poor sleep quality, etc. and can lead to exacerbation of psychiatric symptoms, such as hallucinations, delusions, and cognitive impairment [32]. Recent studies suggested that frequent napping was linked with multiple adverse outcomes, including depression [33], increased mortality [9] and cognitive decline [33], implying the directionality of daytime napping and adverse outcomes is important. In a study of the sleep habits of non-hospitalized middle-aged men and women with schizophrenia, it was demonstrated that daytime napping was more frequent in schizophrenia patients compared to healthy controls, and reflected a circadian rhythm disturbance in patients with schizophrenia [34]. Studies indicated that schizophrenia patients have defects in the sleep spindle, while daytime napping can efficiently estimate nocturnal sleep spindle density in schizophrenia patients [35, 36]. Our findings can provide a basis for daytime napping in the intervention and treatment of schizophrenia.

The MR approach can effectively reduce the bias due to confounding factors and unknown reverse causal associations, which were difficult to eliminate in traditional observational studies. Sensitivity analysis also provided consistent results, increasing the robustness and credibility of the conclusions. The current study still has limitations. Firstly, even though the MR method was able to avoid confounder influences and control horizontal pleiotropy through multiple sensitivity analyses, there are still potential and unknown confounding factors and horizontal pleiotropy that could affect the study's results. Secondly, the more frequent daytime napping was a binary exposure, so we were not able to precisely calculate the GWAS data on frequent napping to specific hours or minutes. Thirdly, our study may not be effectively extended to other populations as the exposure GWAS and outcome GWAS were selected from European populations and cannot be extended to Asian populations or other populations at this time. Fourthly, participants in the UK Biobank and 23andMe cohorts were healthier, so our conclusions cannot be generalized to patients with other diseases for now. Finally, there are currently few independent randomized controlled trials on more frequent daytime napping and schizophrenia; the results need to be validated in future clinical trials.

## Conclusion

The findings of this bidirectional two-sample Mendelian randomization study suggested a bidirectional causal association between more frequent daytime napping the occurrence of schizophrenia, implying that daytime napping frequency is a potential intervention for the progression and treatment of schizophrenia.

## Supplementary Information

**Additional file 1:** **Table S1** and **Table S2**.

## Data Availability

The datasets generated and analyzed during the current study are included in *Genetic determinants of daytime napping and effects on cardiometabolic health* [including its supplementary information files, https://doi.org/10.1038/s41467-020-20585-3], and are available in the IEU OpenGWAS project database [https://gwas.mrcieu.ac.uk/] and Psychiatric Genomics Consortium [https://www.med.unc.edu/pgc/].

## Abbreviations

MR: Mendelian randomization
GWAS: Genome-wide association studies
SNP: Single nucleotide polymorphism
IV: Instrumental variable
PGC: Psychiatric genomics consortium
LD: Linkage-disequilibrium
OR: Odds ratio
CI: Confidence interval
IVW: Inverse variance weighted
MR-RAPS: Mendelian randomization robust adjusted profile score
MR-PRESSO: Mendelian randomization pleiotropy residual sum and outlier
